# Establishing Exercise Programs in Rural Settings Through Collaboration With Family Physicians

**DOI:** 10.7759/cureus.38814

**Published:** 2023-05-10

**Authors:** Ryuichi Ohta, Chiaki Sano

**Affiliations:** 1 Community Care, Unnan City Hospital, Unnan, JPN; 2 Community Medicine Management, Shimane University Faculty of Medicine, Izumo, JPN

**Keywords:** pdsa cycle, japan, rural area, exercise program, quality of life, frailty

## Abstract

In healthcare, preventing acute deterioration in activities of daily living (ADL) and quality of life is critical since sustaining ADL can lead to a healthy and joyful life. Frailty is a risk factor for being unable to maintain ADL, and constant exercise is essential for older adults to prevent the progression of frailty. In rural contexts, older people’s frailty is prevalent. We suggested a method for providing exercise programs in collaboration with family physicians in rural settings, respecting the characteristics of rural older people. The concrete implementation was established based on the ecological model and stakeholder analysis. Four plan, do, study, and act cycles were discussed through collaboration with various professionals. For the implementation and sustainability of rural exercise programs, gradual and logistic planning and progression are critical. Family physicians can be one of the drivers for the smooth implementation of rural exercise programs based on the social assessment and ecological model.

## Introduction

The prevention of acute deterioration in activities of daily living (ADL) and quality of life (QOL) is critical [1]. Sustaining ADL can lead to healthy and joyful lives [2]. Frailty, a risk factor for the deterioration of ADL [3], can be caused by aging, reduced exercise, and a sedentary lifestyle. Frailty is prevalent among older patients in many developed countries, including Japan [2]. Frailty interventions, which typically involve regular exercise, are essential in communities. In this study, we observed an older Japanese female from a rural community as an archetypal model of frailty [4]. Concrete intervention methods against frailty are necessary to prevent it in rural communities.

Reducing frailty is essential for maintaining ADL and improving QOL [2]. Frailty among older rural patients is a public health issue. Previous research has shown that effective exercise education and participation in exercise programs can reduce frailty in older adults [1]. This study was a clustered randomized trial to improve physical activity among middle-aged and older community-dwelling people (aged 40-79 years) in rural communities with a community-oriented exercise program driven by community workers who completed local government training. For the effective implementation, continuity, and quality improvement of rural exercise programs, social assessment using various frameworks and establishing quality improvement plans based on the assessment is essential. However, research is lacking in investigating social assessment and establishing concrete methods for implementing rural exercise programs. Patient and physician relationships can be more vigorous in rural contexts than in urban contexts. Patients tend to follow the physicians’ suggestions compared to the suggestions of family and community workers. This trend can be used for implementing rural exercise programs [5]. So, this study aims to clarify the concrete method of implementation with the concrete steps of rural exercise programs based on the framework of PDSA (plan, do, study, and act).

## Technical report

This technical report suggested concrete interventions for rural frailty involving rural families, respecting the present condition of frailty and characteristics of older people in rural contexts to promote exercise implementation of rural exercise programs. The implementation methods are analyzed and clarified based on the ecological model and stakeholder analysis. The discussion section delineates the concrete way of implementing the PSDA cycle framework.

Intervention plan based on the trend of the attitude of older people toward medicine

**Figure 1:**
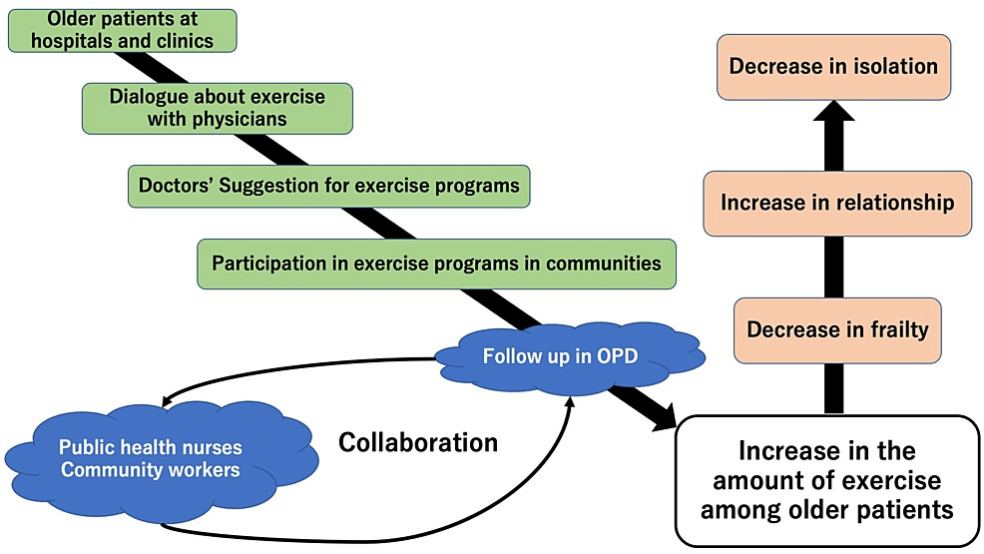
The conceptual framework of the intervention for frailty collaborated with community workers and public health nurses OPD, outpatient department

Based on the trend of the attitude of older people in rural communities, our intervention involves rural family physicians recommending community exercise programs in their outpatient departments (OPDs) for older patients [5]. Community exercise programs are managed by community workers and supported by public health nurses. During follow-up medical visits, rural family physicians asked patients about participation in community exercise programs. The programs can be revised in collaboration with community workers and public health nurses. The intervention flow is shown in Figure 1.

Ecological model and multi-layer intervention in rural communities

This intervention should be conducted using an ecological model. An ecological model is critical for effective community intervention and is focused on solving four levels of public health issues: individual, relational, communal, and social [6]. Multi-layer interventions are more effective than single-layer interventions for tackling communal health problems [7]. Reviewing previous articles, several approaches have been considered based on the four layers of the ecological model.

Regarding the layer of individual and relationship, older patients can improve their muscles and balance with training [8]. Awareness of frailty and early intervention can reduce the risk of frailty and fractures by at least 9.1% in a year [9]. Many older people living alone in rural communities can continuously communicate and understand each other’s physical conditions and activities to prevent frailty [10]. They can establish social relationships in their communities [10] by participating in communal exercise groups. Thus, recommendations to participate in local exercise programs by physicians in rural hospitals can be beneficial. Besides, community workers encourage participants' interactions in local exercise programs in community centers.

Regarding the layer of community, local governments can provide appropriate information and programs regarding effective exercise for preventing frailty, which can improve citizens’ physical activities by 4.6% in five years [1]. So, local governments can provide information about local exercise programs to citizens.

Regarding the layer of society, aging societies increase diseases and put pressure on social health insurance in Japan, so ageism and neglecting older people affect the perception of older patients [11,12]. Revising images can motivate older people to partake in preventive activities and reduce frailty [12]. Thus, physicians, nurses, public health nurses, and staff of local governments provide positive information about aging.

Measures and interventions used in the intervention

Based on an ecological model, the intervention in each layer should be assessed using process and outcome measurements. Table 1 lists the assessment measurements for each layer.

**Table 1:** Multi-layer interventions in rural communities

Layers	Outcome assessment	Process assessment
Individual	- Older patient’s participation rate in exercise programs - Score of loneliness [13]	- Patient's perception of the exercise programs - Medical professionals’ recommendation rate to patients regarding local exercise programs
Relationship	- The number of participants in local exercise programs	- Local exercise program participant's perception of the exercise programs
Community	- The number of community workers working in the exercise programs - The number of times information regarding exercise programs is provided to the communities	- Community workers' perception of the exercise programs
Societal	- Patient's perception of ageism	- The perception of ageism by physicians, nurses, public health nurses, and staff of local governments

Local stakeholders, possible project team members, and stakeholder analysis

Multiple stakeholders should be involved in exercise programs and can manage the balance between their benefits and challenges. The main stakeholders in our project were local government workers, public health nurses, community workers, patients, patient’s families, and medical professionals such as doctors and nurses. Each stakeholder group had different benefits and faced different challenges when implementing the collaborative exercise programs.

Regarding local governments, decreasing frailty can benefit community health and reduce medical expenditure among older people as a benefit [14]. On the other hand, the education of community workers has considerable expenses from the government’s budget. Regarding public health nurses, by increasing rural exercise programs in communities, their resources in health management among older people may increase and can work efficiently [15]. They can also suggest the exercise program to various people. Their present activities for exercise improvement may be similar to these exercise programs, and they may fear their role in health promotion will be diminished.

Motivated community workers can effectively contribute to reducing frailty in their communities by promoting these exercise programs [16]. However, there are different motivations among community workers. The promotion of these exercise programs can induce conflicts among community workers.

Patients and families can find a place to train their bodies for free and reduce frailty. Improving frailty can mitigate the burden of caring for older patients. On the other hand, older patients must try to exercise and adjust their schedules. Their families need time to transfer patients to community centers.

Physicians and nurses can realize the effectiveness of the exercise program by examining their patients. They can also help improve older patients' frailty by encouraging them to participate in the exercise program. However, they need time to collaborate with various stakeholders to allocate patients to the exercise program effectively. They may become busier because of adding the role of facilitating patients.

## Discussion

This technical report clarified the concrete interventional methods of rural exercise programs from the ecological model and stakeholder analysis in rural contexts. PDSA cycles are essential to implement rural exercise programs effectively. In this discussion, the concrete PSDA cycles are discussed based on the contents of the technical report.

Top-down and bottom-up approaches are essential to improve this exercise project. Each stakeholder's motivation and feasibility for this project should be discussed through dialogue. Four PDSA cycles were needed for this project to progress.

The first cycle should involve community workers who educated and facilitated the exercise. Motivation among community workers is essential because they educate patients about exercise [17]. Their perceptions of the project should be clarified to establish a feasible implementation. Perception of frailty and implementation of exercise projects in communities is evaluated through focus group interviews. Through analysis of the interview contents, the feasible exercise projects and ways of implementation can be planned.

The second cycle involved clerks and public health nurses working for local governments. An implementable plan can be constructed through dialogue. Revision of the project based on the discussion with community workers should be performed through discussions with clerks and public health nurses in the local government [18]. The plan can be discussed with clerks and public health nurses in the local government to get support for the project. The hospital physicians and nurses are informed about the project and recommended to inform their patients about it. By analyzing the content of interviews, feasible exercise projects and ways of implementation are planned.

The third cycle involved family physicians, nurses, and clerks. These individuals can collaborate to invite patients to exercise programs through dialogue effectively. Establishing the flow of frail patients to exercise projects can be driven by community workers [18]. The opinions of hospital family physicians, nurses, and clerks are integral for establishing adequate flow. By collaborating with them, the prototype of the flow of patients to the exercise programs is established. The feasible flow of patient invitations to exercise programs and the ways of implementation are planned.

The fourth cycle can involve implementing the established program of inviting older patients to exercise programs and improving their amount of exercise. The primary outcome can be measured six months after the implementation as the rate of older patients in the rural hospital participating in the exercise programs. The program can be implemented for three months based on the flow. The participants and community workers are interviewed to revise the programs. Measuring the number of older patients participating in programs in a rural hospital and their loneliness score [13]. Thematic analysis can be performed based on the interviews with the participants and community workers in the exercise program to revise the program.

## Conclusions

Rural community-based exercise programs collaborating with family physicians can benefit the issue of frailty in rural communities. Through the analysis of the ecological model and stakeholder analysis, community members’ motivations should be respected, and substantial support from local governments and hospitals can be needed. Discussions with various stakeholders, including medical professionals, community workers, and local governments, are essential for rural community-based exercise programs. Furthermore, based on the program's qualitative and quantitative analysis, PDSA cycles should be progressed for the project's continuity and sustainability.
